# Prevalence of Myopia and Its Related Factors Among University Students in Madinah, Saudi Arabia

**DOI:** 10.7759/cureus.49656

**Published:** 2023-11-29

**Authors:** Hanan Makhdoum, Ahmed Alrehaili, Ahmed Albelowi, Ghaida H Aljabri, Ruba A Alamri, Bushra Alawfi, Saba Alsaedi, Reem A Garah

**Affiliations:** 1 Department of Ophthalmology, College of Medicine, Taibah University, Madinah, SAU; 2 College of Medicine, Taibah University, Madinah, SAU

**Keywords:** university students, near-sightedness, refractive error, prevalence, myopia

## Abstract

Background: Myopia, a common refractive error, is a growing global health burden influenced by both genetic and environmental factors. Despite its high prevalence, studies on its prevalence and risk factors among university students are lacking.

Objectives: The objective of this study is to investigate the prevalence of myopia and its associated factors among college students in Saudi Arabia's Madinah region.

Methods: A cross-sectional study was conducted in Al-Madinah, Saudi Arabia, from February to June 2023, utilizing a survey that was distributed to college students through a social media application.

Results: A total of 433 university students from Al-Madinah province were enrolled in this study; 66.3% were females and 33.7% were males. Participants’ ages ranged from 18 to 33 years with a mean of 21.3 ± 2.0 years. The prevalence of myopia among college students in Al-Madinah and its provinces was 57.3%, and 87.9% of them had myopia in both eyes. Respondents with an electronic screen time of more than three hours and a reading distance of less than 15cm were at significant risk of myopia with p-values of 0.037 and 0.019, respectively.

Conclusions: A significant prevalence of myopia has been observed among university students in Madinah. Studying in scientific and medical fields, having eye diseases, prolonged use of digital devices, limiting daily outdoor activities to one hour, and having a reading distance of less than 15 cm significantly increased the risk of myopia. Encouraging education and screening programs for myopia prevention and control is crucial.

## Introduction

Myopia, often known as nearsightedness or short-sightedness, is a spherical refractive defect that causes the eye to fail to see distant objects [1]. It is caused by high refractive power relative to corneal curvature and lens thickness and increased anteroposterior eyeball diameter, resulting in light refraction to a focal point in front of the retina [2,3].

Myopia is the most common refractive disease, particularly in adolescents and young adults [4]. Its rapidly increasing prevalence and incidence worldwide in recent decades have sparked substantial international concern. Myopia is expected to affect 49.8% of the global population by 2050 [5]. The current incidence of myopia in Saudi Arabia is 48.7% among adults in Riyadh [6] and 53.5% among college students in both Hail and Riyadh [7]. Myopia manifests clinically as blurry distance vision, eye rubbing, and squinting [8]. Myopia is considered a major global public health concern and has become a leading cause of vision impairment.

Myopia’s exact cause remains unknown despite numerous studies that have attempted to explain it. However, myopia development and progression have been associated with several genetic and environmental risk factors. Several studies have associated having myopic parents with an increased likelihood of developing the condition [9-11]. Younger age at myopia onset is thought to be a risk factor for myopia advancement [12]. Other studies have found that myopia may be influenced by environmental factors, such as a higher level of education, more near work, and fewer outside activities [13,14]. Furthermore, computer and smartphone use is widespread in daily life, and myopia may develop or worsen due to digital device use [15].

Many factors might protect against myopia. An Australian cohort study found an inverse relationship between myopia and daylight exposure, which remained significant even after adjusting for sex, age, parental history of myopia, and educational level [16]. Many studies have found a positive association between extensive schoolwork and the onset and progression of myopia. This association was explained by near work, including reading and writing [17]. Therefore, reducing the burden of schoolwork, especially during the first educational years, could help prevent myopia.

Many studies have investigated the prevalence, risk factors, and progression of myopia in childhood. However, it remains understudied in adults. Despite having behavioral characteristics related to an increased incidence of myopia, this is especially true among university students. This study aimed to investigate the prevalence of myopia and its associated characteristics among university students in Saudi Arabia’s Madinah region.

## Materials and methods

This cross-sectional observational analytical study was conducted from February to June 2023 in Al-Madinah, Saudi Arabia, using a survey sent via social media application to university students in the Al-Madinah region of Saudi Arabia. Students with ocular disorders such as cataracts, glaucoma, congenital eye issues, and refractive anomalies other than myopia were excluded from this study. The four sections of the pre-designed questionnaire were taken from a prior Chinese study: consent and confidentially, personal data, myopia pattern, and factors related to myopia. Before recruitment, participants were given details about the study’s objectives, including duration and confidentiality. They were also informed that their data would be used for study-related purposes, but their identities would be kept confidential. On April 1, 2023, the Taibah University College of Medicine’s Scientific Research Ethics Committee ethically approved this study (Study ID: TU-021-22-IRB00010413).

All acquired data were coded before being entered into a computer. IBM SPSS Statistics for Windows, Version 28 (Released 2021; IBM Corp., Armonk, New York, United States) was used for data entry and analysis. Categorical variables are presented as frequencies and percentages, and numerical variables are presented as arithmetic means, ranges, and standard deviations (SD). Categorical variables were compared using the Chi-square test. Continuous variables were compared using the independent samples t-test. After correcting for confounding factors, a logistic regression analysis with multivariate variables was used to identify myopia-related factors. Myopia was treated as a dichotomous variable (yes/no), and the results are expressed as adjusted odds ratios (AORs) and 95% confidence intervals (CIs). A p-value of <0.05 was considered statistically significant.

## Results

The characteristics of the 433 university students who responded to the survey are summarized in Table 1. They were aged 18 to 33 years, with a mean of 21.3 ± 2 years. Two hundred and eighty-seven (66.3%) of the participants were female, 413 (95.4%) reside in Medina and 377 (87.1%) of them attend Taibah University. Two hundred and thirty-seven (54.8%) of the participants were enrolled in medical colleges.

**Table 1:** Participants’ characteristics (n = 433).

Personal characteristics	Frequency	Percentage
Gender		
Male	146	33.7
Female	287	66.3
Age in years		
Range	18-33	
Mean±SD	21.3±2.0	
Residency		
Medina	413	95.4
Others	20	4.6
University		
Taibah University.	377	87.1
Alrayan College.	22	5.1
University of Prince Mugrin.	20	4.6
Others	14	3.2
Specialty		
Medical	237	54.8
Scientific	134	30.9
Literature	62	14.3

**Figure 1:**
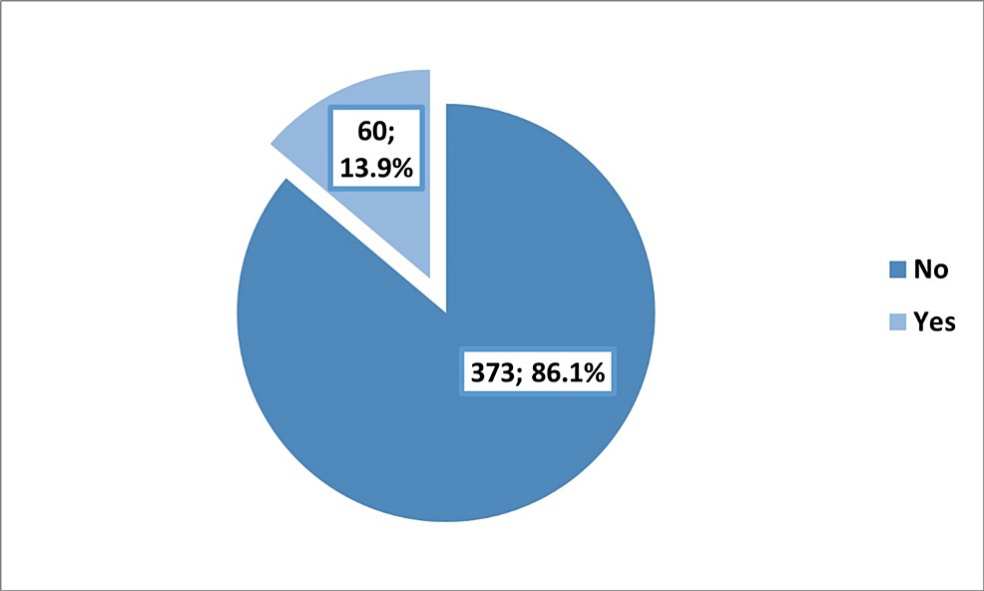
History of any eye disease other than myopia among the participants.

A history of any eye disease other than myopia was reported by 60 (13.9%) of the participants (Figure 1), and a history of any corrective refractive surgery was reported by 15 (3.5%).

Myopia prevalence

Approximately 248 (57.3%) of the participants had myopia; if those who did not know were omitted, the prevalence was 61.7%. Among affected participants, 218 (87.9%) reported that both eyes were affected. Two hundred and fifty-one (58%) of the participants reported wearing visual aids, mainly glasses in 167 (38.6%) or both glasses and lenses in 80 (18.5%) of the participants. One hundred and twenty-seven (29.3%) of the participants reported wearing visual aids all day. Two hundred and forty-four (56.4%) of the participants reported a usual reading distance of <15 cm. Two hundred and eighty-four (65.6%) of the participants reported never having undergone a visual assessment, whereas 120 (27.7%) had them annually. Two hundred and seventy-one (62.5%) of the participants reported a parental history of myopia, of which 123 (28.4%) reported that both parents were affected.

Vision-related behavior

Table 2 shows that 303 (70%) of the participants reported having poor posture when writing or reading and 309 (71.4%) of the participants were not taking breaks after 30 minutes of continuous reading. Three hundred and eighty-three (88.5%) of the participants spent >3 hours using digital devices daily. Two hundred and thirty-two (53.6%) reported having ≤7 hours of sleep. Three hundred and fifty-one participants (81.1%) spent 2-4 hours or more daily doing near work, whereas 186 (43%) spent 1-2 hours outdoors daily. One hundred and fifty-five participants (35.8%) reported a history of weekly exercise, and 68 (15.7%) reported regularly engaging in sports practice. The use of appropriate lighting for studying and practicing eye exercises was reported by 228 (52.7%) and 27 (6.2%) of the participants, respectively, while regular eye washing at night was reported by 82 (18.9%).

**Table 2:** Participants’ vision-related behavior.

Variables	Frequency	Percentage
Having bad posture when writing or reading		
No	130	30.0
Yes	303	70.0
Having the habit of having breaks after 30 minutes of continues reading		
No	309	71.4
Yes	124	28.6
Length of time spent each day using digital device		
None	9	2.1
Less than one hour	4	0.9
1-2 hours	7	1.6
2-3 hours	30	6.9
More than three hours	383	88.5
Average sleep time per day		
Less than or equal to seven hours	232	53.6
More than seven hours	201	46.4
Length of time spent on close work every day		
Less than or equal to two hours	82	18.9
2 – 4 hours	142	32.8
4 – 6 hours	117	27.0
6 – 8 hours	47	10.9
More than eight hours	45	10.4
Length of time spent outdoors every day		
Less than one hour	101	23.3
1 - 2 hours	186	43.0
More than two hours	146	33.7
History of weekly exercise		
No	278	64.2
Yes	155	35.8
Frequency of engaging in sports practice		
None	121	27.9
Irregularly	244	56.4
Regularly	68	15.7
Using appropriate lighting for studying		
No	205	47.3
Yes	228	52.7
Practicing any eye exercises		
No	406	93.8
Yes	27	6.2
Frequency of washing eyes at night		
None	218	50.4
Irregularly	133	30.7
Regularly	82	18.9

Factors associated with myopia

Personal and Medical Factors

In this study, 242 (62.9%) of the participants living in Medina had myopia, compared to 6 (35.3%) of those living outside Medina (p = 0.022). In addition, 143 (65.3%) of participants studying medicine and 81 (64.3%) studying science had myopia, compared to only 24 (42.1%) of participants studying literature (p = 0.004). Moreover, participants who reported a history of any eye disease other than myopia were more likely to have myopia than those who did not (76.3% vs. 59.2%; p = 0.013). Furthermore, participants who reported a usual reading distance of <15 cm were more likely to develop myopia than those who reported a distance of >15 cm (66.2% vs. 55.9%; p = 0.035). Finally, more participants who reported undergoing an annual visual assessment had myopia than those who reported never having such an assessment (88.3% vs. 47.0%; p < 0.001; Table 3).

**Table 3:** Bivariate analysis of personal and medical factors associated with myopia among participants.

	Myopia		p-value
	No	Yes	
	n-154 N (%)	N=248 N (%)	
Gender			
Male (n=133)	54 (40.6)	79 (59.4)	0.506*
Female (n=269)	100 (37.2)	169 (62.8)	
Age in years			
Mean±SD	21.3±1.9	21.3±2.1	0.929**
Residency			
Medina (n=285)	143 (37.1)	242 (62.9)	0.022*
Others (n=17)	11 (64.7)	6 (35.3)	
University			
Taibah University (n=350)	130 (37.1)	220 (62.9)	0.662*
Alrayan College (n=21)	10 (47.6)	11 (52.4)	
University of Prince Mugrin (n=20)	9 (45.0)	11 (55.0)	
Others (n=11)	5 (45.5)	6 (54.5)	
Specialty			
Medical (n=219)	76 (34.7)	143 (65.3)	0.004*
Scientific (n=126)	45 (35.7)	81 (64.3)	
Literature (n=57)	33 (57.9)	24 (42.1)	
History of having any eye diseases, other than myopia			
No (n=343)	140 (40.8)	203 (59.2)	0.013*
Yes (n=59)	14 (23.7)	45 (76.3)	
History of ever having any corrective refractive surgery			
No (n=387)	151 (39.0)	236 (61.0)	0.137*
Yes (n=15)	3 (20.0)	12 (80.0)	
The usual reading distance			
Less than 15 cm (n=225)	76 (33.8)	149 (66.2)	0.035*
More than 15 cm (n=177)	78 (44.1)	99 (55.9)	
Frequency of performing visual assessment			
Never (n=253)	134 (53.0)	119 (47.0)	<0.001*
Every 6 months (n=29)	6 (20.7)	23 (79.3)	
Every year (n=120)	14 (11.7)	106 (88.3)	
Parental history of myopia			
Neither (n=150)	61 (40.7)	89 (59.3)	0.245*
Father (n=75)	27 (36.0)	48 (64.0)	
Mother (n=64)	18 (28.1)	46 (71.9)	
Both (n=113)	48 (42.5)	65 (57.5)	

Vision-Related Behavioral Factors

Participants who spent longer each day using digital devices (>3 hours) were more likely to have myopia than those who never used these devices (61.5% vs. 22.2%; p = 0.037). In addition, participants who spent, on average, <1 hour/day outdoors were more likely to develop myopia than those who spent >2 hours/day outdoors (72.8% vs. 57.2%; p = 0.041; Table 4).

**Table 4:** Bivariate analysis of vision-related behavioral factors associated with myopia among participants.

	Myopia		p-value*
	No	Yes	
	n-154 N (%)	N=248 N (%)	
Having bad posture while reading or writing			
No (n=118)	52 (44.1)	66 (55.9)	0.126
Yes (n=284)	102 (35.9)	182 (64.1)	
Having the habit of having breaks after 30 minutes of continuous reading			
No (n=288)	105 (36.5)	183 (63.5)	0.225
Yes (n=114)	49 (43.0)	65 (57.0)	
Length of time spent each day using digital device			
None (n=9)	7 (77.8)	2 (22.2)	0.037
Less than one hour (n=2)	1 (50.0)	1 (50.0)	
1-2 hours (n=6)	3 (50.0)	3 (50.0)	
2-3 hours (n=29)	6 (20.7)	23 (79.3)	
More than three hours (n=356)	137 (38.5)	219 (61.5)	
Average sleep time per day			
Less than or equal to seven hours (n=211)	85 (40.3)	126 (59.7)	0.392
More than seven hours (n=191)	69 (36.1)	122 (63.9)	
Length of time spent on close work every day			
Less than or equal to two hours (n=79)	29 (36.7)	50 (63.3)	0.110
2 – 4 hours (n=132)	54 (40.9)	78 (59.1)	
4 – 6 hours (n=107)	31 (29.0)	76 (71.0)	
6 – 8 hours (n=41)	19 (46.3)	22 (53.7)	
More than 8 hours (n=43)	21 (48.8)	22 (51.2)	
Length of time spent outdoors every day			
Less than1 hour (n=92)	25 (27.2)	67 (72.8)	0.041
1 - 2 hours (n=172)	70 (40.7)	102 (59.3)	
More than two hours (n=138)	59 (42.8)	79 (57.2)	
History of weekly exercise			
No (n=258)	92 (35.7)	166 (64.3)	0.144
Yes (n=144)	62 (43.1)	82 (56.9)	
Frequency of engaging in sports practice			
None (n=112)	45 (40.2)	67 (59.8)	0.555
Irregularly (n=227)	82 (36.1)	145 (63.9)	
Regularly (n=63)	27 (42.9)	36 (57.1)	
Using appropriate lighting for studying			
No (n=187)	69 (36.9)	118 (63.1)	0.588
Yes (n=215)	85 (39.5)	130 (60.5)	
Practicing any eye exercises			
No (n=379)	146 (38.5)	233 (61.5)	0.720
Yes (n=23)	8 (34.8)	15 (65.2)	
Frequency of washing eyes at night			
None (n=205)	74 (36.1)	131 (63.9)	0.640
Irregularly (n=122)	49 (40.2)	73 (59.8)	
Regularly (n=75)	31 (41.3)	44 (58.7)	

Multivariate logistic regression analysis

Multivariate logistic regression analysis showed that participants with a history of any eye disease other than myopia were at almost threefold higher risk of myopia (AOR = 2.65, 95% CI = 1.29-5.47, p = 0.008, Table 5). In contrast, participants whose usual reading distance was >15 cm were less likely to develop myopia than those whose usual reading distance was <15 cm (AOR = 0.57, 95% CI = 0.36-0.91, p = 0.019). In addition, participants who underwent a visual assessment every six months (AOR = 4.24, 95% CI = 1.60-11.24, p = 0.004) or year (AOR = 10.30, 95% CI = 5.35-19.82, p < 0.001) were at higher risk for myopia than those who had never undergone one. Moreover, participants who used digital devices for 2-3 hours/day were at higher risk for myopia than those who did not use digital devices (AOR = 12.46, 95% CI = 1.67-92.94, p = 0.014). Furthermore, participants who spent 1-2 hours (AOR = 0.43, 95% CI = 0.23-0.81, p = 0.009) or >2 hours (AOR = 0.40, 95% CI = 0.21-0.77, p = 0.006) outdoors were less likely to develop myopia than those who spent <1 hour/day outdoors. Participants’ residency and specialty were not significantly associated with myopia.

**Table 5:** Multivariate logistic regression analysis of determinants of myopia among participants.

	Adjusted odds ratio	95% confidence interval	p-value
History of having any eye diseases, other than myopia			
No^a^	1.0	---	---
Yes	2.65	1.29-5.47	0.008
The usual reading distance			
Less than 15 cm^a^	1.0	---	---
More than 15 cm	0.57	0.36-0.91	0.019
Frequency of performing visual assessment			
Never^a^	1.0	---	---
Every 6 months	4.24	1.60-11.24	0.004
Every year	10.30	5.35-19.82	<0.001
Length of time do spent each day using digital device			
None^a^	1.0	---	---
Less than 1 hour	1.83	0.05-63.41	0.739
1-2 hours	1.12	0.08-15.34	0.931
2-3 hours	12.46	1.67-92.94	0.014
More than 3 hours	5.47	0.96-31.20	0.056
Length of time spent outdoors every day			
Less than1 hour^a^	1.0	---	---
1 - 2 hours	0.43	0.23-0.81	0.009
More than 2 hours	0.40	0.21-0.77	0.006

## Discussion

Our study found a lower percentage of myopic students (57.3%) than earlier studies. A Chinese study by Huang et al. involving 1153 university students in Nanjing found myopia prevalence to be 86.8% [18]. This difference might be explained by Asian individuals being more prone to developing myopia, leading to its higher prevalence in Asian populations in various studies [19,20]. An observational study by Alamri et al. evaluating myopia prevalence among King Khalid University medical students reported that almost half of the participants were myopic [3]. Our result is consistent with their finding since we found that about two-thirds of medical students (65.3%) had myopia. In addition, another study on Norwegian medical students supports our findings of higher myopia prevalence among medical students [21]. This increased myopia prevalence might be explained by individuals with higher educational attainment and academic achievement spending significantly more time on near-work activities such as reading with less outside activity [22].

Almost two-thirds (62.5%) of our participants reported a family history of myopia, of which 28.4% reported that both parents were affected. Other studies have suggested that having parents with myopia may increase the likelihood of developing myopia [18,23], implying a hereditary susceptibility to myopia.

More than half of our participants engaged in near work on screens. In addition, participants who typically read from a <15 cm distance were more likely to develop myopia than those who read from a >15 cm distance (66.2% vs. 55.9%). A Chinese study and an Australian study found that children who engage in near work had a higher risk of developing myopia [24,25]. Our study suggests that continuous reading for >30 minutes increases the risk of myopia, possibly due to increased accommodative lag. Additionally, Dutheil et al. found that near work led to a higher myopia prevalence in adults, with the odds of myopia development increasing to 21% [26]. Regardless, in many other studies, this risk has not been identified [13,18]. The association between near work and myopia development and progression requires further evidence-based research.

A Chinese study in Nanjing concluded that taking breaks after continuous reading decreased the likelihood of developing myopia [27], indicating that this behavior is protective. Our study found similar protection. Our study did not find that eye exercise and eye washing were protective, consistent with the findings of the King Khalid University study [3].

Our study showed that students who spent 2-3 hours or more/day using digital devices were at increased risk of myopia. These results are consistent with a Spanish cohort study on 17,218 university students that found exposure to digital screens led to myopia development and progression [15]. Nonetheless, there is much controversy regarding the relationship between myopia and exposure to digital devices, which is based on inconsistent findings from various studies and a systematic review by Lanca et al. [3,7,28]. Therefore, additional studies are needed to better clarify their relationship.

Our study found that students who participated in outdoor activities for >2 hours were less likely to develop myopia. This finding likely reflects the inhibition of eye growth due to retinal dopamine release in response to exposure to higher light intensities in outdoor environments [29]. Our finding is consistent with many research studies, including a cohort study on children aged 10-15 years, where the protective effects of outdoor activities led to less axial eye growth [30].

One limitation of our study was its use of a self-administered questionnaire, which risks subjective and recall bias. It also used a non-probability sampling method (convenience), which risks a non-representative sample. Using a validated and reliable questionnaire is one of our study’s strengths. Furthermore, our selected population was academic students who could better understand the survey questions and terminology. Furthermore, 50% were from medical science colleges and are, to some extent, knowledgeable about their condition (i.e., minimizing understanding errors that could occur from the general population due to educational background).

Recommendations

Interventions that promote visual health and reduce myopia in society are desperately needed because it is a severe public health problem among children and adolescents. Therefore, additional education and screening programs for children should be encouraged to prevent and control myopia. The outcomes of our study may be crucial for visual health by identifying factors associated with myopia. The findings of our study may increase students’ awareness of the importance of visual health and contribute to the creation of future vision health policies. More research is needed to better understand myopia’s prevalence and risk factors. Future studies should strive for larger sample sizes, more cities in Saudi Arabia, and better study design.

## Conclusions

In conclusion, our study revealed that myopia affected more than half of the university students in Madinah, Saudi Arabia. According to our findings, many factors were found to increase the risk of myopia some of which are family history, near reading, and reduced outdoor activities. There is a great need for myopia screening programs and education in order to control it and combat its progression due to its significantly high public health burden.

## References

[1] Parveen N, Hassan SN, Rehman J (2019). Prevalence of myopia and its associated risk factors in local medical students. Medical Channel.

[2] Fredrick DR (2002). Myopia. BMJ.

[3] Alamri AR, Al Kaabi HA, Al Jallal MS (2022). Prevalence of myopia among medical students in King Khalid University and its effects on academic performance. Bahrain Med Bull.

[4] Chiang SY, Weng TH, Lin CM, Lin SM (2020). Ethnic disparity in prevalence and associated risk factors of myopia in adolescents. J Formos Med Assoc.

[5] Holden BA, Fricke TR, Wilson DA (2016). Global prevalence of myopia and high myopia and temporal trends from 2000 through 2050. Ophthalmology.

[6] Almudhaiyan T, Alhamzah A, AlShareef M (2020). The prevalence of refractive errors among Saudi adults in Riyadh, Saudi Arabia. Saudi J Ophthalmol.

[7] Ahmed HG, Algorinees RM, Alqahtani NT, Aljarbou M, AlShammari RS, Alrashidi AG, Alshammari BT (2019). Prevalence of myopia and its related risk factors among medical students in Saudi Arabia. Adv Ophthalmol Vis Syst.

[8] Saw SM, Katz J, Schein OD, Chew SJ, Chan TK (1996). Epidemiology of myopia. Epidemiol Rev.

[9] Jones-Jordan LA, Sinnott LT, Manny RE (2010). Early childhood refractive error and parental history of myopia as predictors of myopia. Invest Ophthalmol Vis Sci.

[10] Pacella R, McLellan J, Grice K, Del Bono EA, Wiggs JL, Gwiazda JE (1999). Role of genetic factors in the etiology of juvenile-onset myopia based on a longitudinal study of refractive error. Optom Vis Sci.

[11] Kurtz D, Hyman L, Gwiazda JE, Manny R, Dong LM, Wang Y, Scheiman M (2007). Role of parental myopia in the progression of myopia and its interaction with treatment in COMET children. Invest Ophthalmol Vis Sci.

[12] Hyman L, Gwiazda J, Hussein M, Norton TT, Wang Y, Marsh-Tootle W, Everett D (2005). Relationship of age, sex, and ethnicity with myopia progression and axial elongation in the correction of myopia evaluation trial. Arch Ophthalmol.

[13] Lin Z, Vasudevan B, Jhanji V (2014). Near work, outdoor activity, and their association with refractive error. Optom Vis Sci.

[14] Huang L, Kawasaki H, Yasuda R, Sakai R (2018). Relationship between visual acuity and lifestyle: a cross-sectional study in Japanese children. Hiroshima J Med Sci.

[15] Fernández-Montero A, Olmo-Jimenez JM, Olmo N, Bes-Rastrollo M, Moreno-Galarraga L, Moreno-Montañés J, Martínez-González MA (2015). The impact of computer use in myopia progression: a cohort study in Spain. Prev Med.

[16] McKnight CM, Sherwin JC, Yazar S (2014). Myopia in young adults is inversely related to an objective marker of ocular sun exposure: the Western Australian Raine cohort study. Am J Ophthalmol.

[17] Huang HM, Chang DS, Wu PC (2015). The association between near work activities and myopia in children - a systematic review and meta-analysis. PLoS One.

[18] Huang L, Kawasaki H, Liu Y, Wang Z (2019). The prevalence of myopia and the factors associated with it among university students in Nanjing: a cross-sectional study. Medicine (Baltimore).

[19] Kleinstein RN, Jones LA, Hullett S (2003). Refractive error and ethnicity in children. Arch Ophthalmol.

[20] Saw SM, Pan CW, Dirani M, Cheng CY, Wong TY (2014). Is myopia more common in Asians? A systematic review and meta-analysis. Invest Ophthalmol Vis Sci.

[21] Midelfart A, Aamo B, Sjøhaug KA, Dysthe BE (1992). Myopia among medical students in Norway. Acta Ophthalmol (Copenh).

[22] Mutti DO, Mitchell GL, Moeschberger ML, Jones LA, Zadnik K (2002). Parental myopia, near work, school achievement, and children’s refractive error. Investig Ophthalmol Vis Sci.

[23] Wang L, Du M, Yi H (2017). Prevalence of and factors associated with myopia in inner Mongolia medical students in China, a cross-sectional study. BMC Ophthalmol.

[24] Lin Z, Vasudevan B, Mao GY (2016). The influence of near work on myopic refractive change in urban students in Beijing: a three-year follow-up report. Graefes Arch Clin Exp Ophthalmol.

[25] Ilhan N, Ilhan O, Ayhan Tuzcu E, Daglioglu MC, Coskun M, Parlakfikirer N, Keskin U (2014). Is there a relationship between pathologic myopia and dry eye syndrome?. Cornea.

[26] Dutheil F, Oueslati T, Delamarre L (2023). Myopia and near work: a systematic review and meta-analysis. Int J Environ Res Public Health.

[27] Zhou Y, Huang XB, Cao X (2022). Prevalence of myopia and influencing factors among high school students in Nantong, China: A cross-sectional study. Ophthalmic Res.

[28] Lanca C, Saw SM (2020). The association between digital screen time and myopia: a systematic review. Ophthalmic Physiol Opt.

[29] McCarthy CS, Megaw P, Devadas M, Morgan IG (2007). Dopaminergic agents affect the ability of brief periods of normal vision to prevent form-deprivation myopia. Exp Eye Res.

[30] Read SA, Collins MJ, Vincent SJ (2015). Light exposure and eye growth in childhood. Invest Ophthalmol Vis Sci.

